# Novel humidity sensors based on nanomodified Portland cement

**DOI:** 10.1038/s41598-021-87563-7

**Published:** 2021-04-14

**Authors:** Thanyarat Buasiri, Karin Habermehl-Cwirzen, Lukasz Krzeminski, Andrzej Cwirzen

**Affiliations:** 1 Building Materials, Department of Civil, Environmental and Natural Resources Engineering, Luleå University of Technology, 97187 Luleå, Sweden; 2 The Institute of Engineering Materials and Biomaterials, Silesian University of Technology, 44-100 Gliwice, Poland

**Keywords:** Engineering, Materials science, Nanoscience and technology

## Abstract

Commonly used humidity sensors are based on metal oxides, polymers or carbon. Their sensing accuracy often deteriorates with time, especially when exposed to higher temperatures or very high humidity. An alternative solution based on the utilization of Portland cement-based mortars containing in-situ grown carbon nanofibers (CNFs) was evaluated in this study. The relationship between the electrical resistivity, CNF content and humidity were determined. The highest sensitivity was observed for samples containing 10 wt.% of the nanomodified cement which corresponded to 0.27 wt.% of CNFs. The highest calculated sensitivity was approximately 0.01024 per 1% change in relative humidity (RH). The measured electrical resistivity is a linear function of the RH in the humidity range between 11 and 97%. The percolation threshold value was estimated to be at around 7 wt.% of the nanomodified cement, corresponding to ~ 0.19 wt.% of CNFs.

## Introduction

Humidity sensing plays an important part in, e.g. production processes or building maintenance. Cement hydration is controlled by moisture content and thus humidity^1^. Uncontrolled or improper moisture diffusion, especially in an early stage of hydration, can result in a number of negative effects. These include increased shrinkage, lower long-term strength and durability problems^2, 3^. An insufficient water content, e.g. related to an excessive evaporation, can hinder the hydration process^4^. Therefore, monitoring the humidity is crucial for concrete technology. The humidity defined as the amount of water vapour present in the gas phase and can be expressed as absolute or relative value. The relative humidity (RH) is the ratio between the measured amount of water vapour and the water vapour required to reach the saturation state at a certain temperature. Measuring the mass of water vapour contained in a unit volume determines to the absolute humidity (AH).

Humidity measurements rely on converting the detected amount of water molecules into a signal that can be measured, analysed, interpreted and quantified. The interaction between water molecules and sensors is controlled by various physical phenomena. The physical phenomenon being measured defines which type of sensor is used. The most conventional types include capacitive, resistive, impedance, quartz crystal microbalance (QCM), optic-fiber, surface acoustic wave (SAW) or resonance sensing^5–7^. The capacitive sensors are the most commonly used with an estimated 75% of the total market share^8^. They are built of two metal plates separated by a thin layer of an non-conductive polymer film^9^. The non-conductive polymer film attracts moisture from the air, which changes the dielectric constant of the hygroscopic layer. Various types of materials have been used for the moisture sensitive layer. The most common include for example polyimide film (DuPont 5878), polymethyl methacrylate (PMMA), porous ceramics, porous silicon, porous silicon carbide, hygroscopic polymers or porous alumina (Al_2_O_3_ )^8, 10, 11^. Capacitive sensors tend to have a variable sensitivity depending on the measured humidity levels. For example sensors using plasma-etched polyimide as the sensing layer showed low sensitivity at a RH up to 70% and very high sensitivity at a RH between 70 and 90% ^11^. Similar problems were observed when using porous silicon. In that case, hysteresis was observed at a RH above 60%^9^. The required power demand is rather low but the production technology of this type of sensors is complicated^12^. Resistive sensors overcame some of the problems typical for the capacitive sensors. They also are easier and cheaper to fabricate, have high sensitivity and low power consumption. Metal oxide, polymers, and carbon-based materials are the most frequently used materials for their production. Unfortunately, some of these materials degrade when exposed to high humidity, including for example, metal oxide or polymer sensors^13^. Slow response/recovery time and high operating temperatures remain the main design challenges for these sensors. Sensors based on matrixes containing Portland cement measure also changes of resistivity^14^. In this case, the measured changes were related to alterations of the pore structure leading to either shrinkage or expansion of the matrix, water absorption or desorption hysteresis at the interface. Humidity sensing of such matrixes depends also on the amount of the incorporated conductive material like carbon fibers (CFs). Chen et al.^15^ revealed that cementitious matrixes containing less CFs better sense changes of the internal humidity. Different curing conditions are known to affect the sensing capability as well. Sun et al.^16^ reported that a cementitious matrix reinforced with CFs cured in air had a more than 2-times lower electrical resistivity compared to the same material being oven cured. Results obtained by Han et al.^17^ showed the same trend. Optic fiber humidity sensors can be classified according to their working principles including optical absorption of materials, optical fiber Bragg gratings (FBG), interferometric method, and evanescent wave. The FBG-based sensors have been used to monitor strain and temperature^18–20^ as well as humidity in highways^19^. FBG uses a permanent periodic modulation of the reflective index which is formed by an exposure of the core of the optical fiber to an intense optical interference pattern of the light^21^. The humidity sensing is based on an interaction of water molecules with the sensitive core layer. This results in a change of the effective refractive index of the fiber core and in a shift of the Bragg wavelength^22^. Several polymeric materials such as polymide^23, 24^, di-ureasil^21^ and PMMA^25, 26^ have been coated onto an etched FBG to improve the humidity sensitivity. Etching of cladding and coating with graphene oxide^27^ or CNTs^22^ layer showed sensitivity of ∼31 pm/%RH. Furthermore, the modified sensor could detect the relative humidity over a wide range between 20 and 90% at a constant temperature of 25 ˚C^22^. PMMA-based microstructure polymer optical fiber Bragg were studied by Woyessa et al.^28^. The results showed a response having a very low hysteresis and an improved humidity sensitivity (∼35 pm/%RH at 90% RH).

Yet, carbon-based materials are widely used to induce the sensing capability into the monitored material. For example, Chung et al.^29–32^, Sun et al.^33^ and Ou et al.^34^ induced piezoresistive properties of cementitious composites by incorporating CFs. Camacho-Ballesta et al.^35^ reported that composites containing only 0.05 wt.% of CNTs showed electrical properties sufficient to be used for monitoring. Yu and Kwon^36^ revealed CNT/cement composites have high sensitivity of the composite stress response when the CNT doping level is high. Composites based on Portland cement with directly grown CNFs showed even better results in stress/strain monitoring reaching approximately 90% compared with the conventional cementitious material^37^. CNTs and CNFs are commonly added as aqueous dispersions. Unfortunately, their strong hydrophobicity results in a formation of agglomerates. The effect is even stronger in high pH solutions present during the Portland cement hydration. The formation of agglomerates prevents the uniform distribution of the fibers throughout the binder matrix, which is the key condition to create an effective electrically conductive network. Surface functionalization of CNTs and CNFs enhanced the mechanical properties of cement matrixes through a better bond but it also increased the agglomeration^38^. The usage of ultra-sonication in the presence of surfactants produced stable suspensions of well dispersed MWCNTs and functionalized CNTs/CNFs^38, 39^. However, the results showed also that an increasing amount of CNTs/CNFs tends to worsen the fresh mix workability.

The development of an alternative technology enabling to synthesize CNFs directly on cement have limited some of these problems. This method uses a chemical vapour deposition process to grow CNFs directly on cement particles by utilizing naturally embedded Fe and Al as catalysts^37, 40, 41^. Replacing part of Portland cement with this nanomodified cement ensured a uniform distribution of CNFs. It mitigated also the loss of workability and thus significantly increased the maximum amount of CNFs, which could be incorporated into the binder matrix. This method seems to be potentially beneficial to create electrically conductive matrixes sensitive in many aspects including humidity variations. The research described in the present paper focused on investigating potential applicability of nanomodified cement to manufacture sensors for humidity measurements.

## Materials and methods

**Figure 1:**
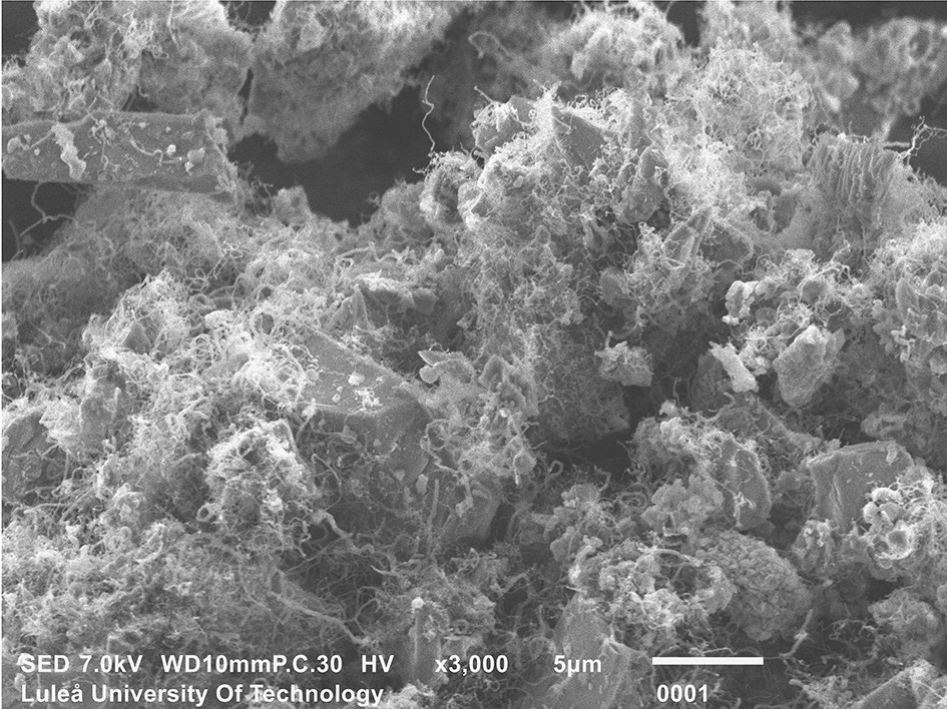
Scanning Electron Microscope image of the nanomodified cement. This figure was taken using InTouchScope software version JSM-IT100 (https://www.jeol.co.jp/en/products/detail/JSM-IT100.html).

Test sensors were produced as mortar beams composed of a mixture of an ordinary Portland cement (OPC) type CEM I 42.5 provided by Cementa-Sweden and the so-called SmartCem. The SmartCem is a nanomodified Portland cement having CNFs synthesized directly on the surface of pristine cement through Chemical Vapour Deposition (CVD). The total amount of carbon nanofibers (CNFs) grown on the SmartCem was approximately 2.71 wt.%. The used synthesis processes is described in detail elsewhere^37^. The morphology of grown CNFs is shown in Fig. 1.

Sieved and clean sand with the maximum particle size of 150 μm was used as fine aggregate. The superplasticizer (sp) type Glenium produced by Grace Chemical with a solid content of 30% was used to control the workability of the fresh mix. The water-to-binder ratio and the sand-to-binder ratio (s/b) were constant at 0.35 and 1 respectively. The mix proportions used for the test mortars are shown in Table 1. Three samples were produced for each mix and measured electrical resistivity showed less than 5% variation. Measurements also indicated that 900 s waiting time was required to obtain a stable reading. All measurements were done after that waiting period.

**Table 1:** Mix proportions.

Mix	w/b	s/b	sp	Cement	Nanomodified cement (SmartCem)	Amount of CNFs
			(wt.% of binder)	(wt.% of binder)	(wt.% of binder)	(wt.% of binder)
Ref	0.35	1.0	0.8	100	0	0.000
S2	0.35	1.0	0.8	98	2	0.054
S4	0.35	1.0	0.8	96	4	0.108
S6	0.35	1.0	0.8	94	6	0.163
S8	0.35	1.0	0.8	92	8	0.217
S10	0.35	1.0	0.8	90	10	0.271

**Figure 2:**
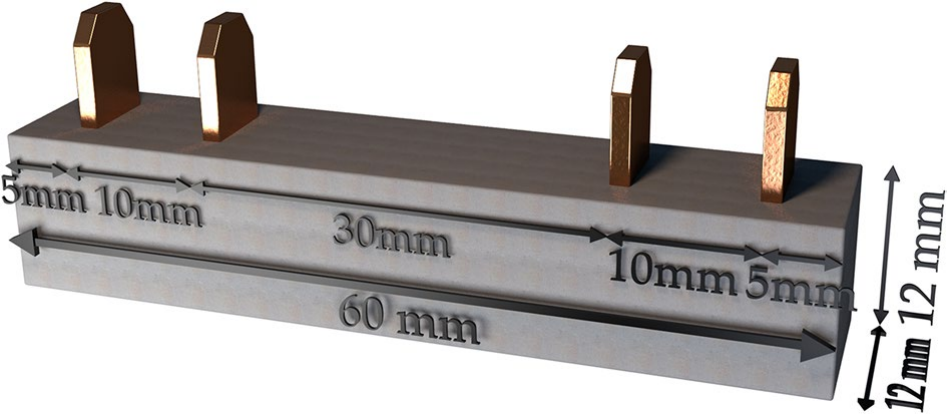
Mortar specimen with four electrodes. This figure was created using Sketchup version 2017 (https://www.sketchup.com), Microsoft PowerPoint version Microsoft 365 (https://www.microsoft.com/en-us/microsoft-365/powerpoint).

Mortars were mixed using a Bredent vacuum mixer and poured into Teflon moulds. The size of test specimens in this research was selected having dimensions of 12 mm × 12 mm × 60 mm since no standard size setup test for electrical resistance measurement at present. Different specimen geometries can be used; however, the measured resistance should be converted to resistivity by using an appropriate geometry correction factor. Four copper electrodes having dimensions of 5 mm × 15 mm × 0.25 mm were immersed 7.5 mm and 30 mm apart into these samples and connected by electrical wires with the measuring system, Fig. 2.

The effects of the curing conditions on the electrical resistivity were determined a using reference sample (Ref) and the mix containing 4 wt.% of the SmartCem (S4). After casting, the samples were cured at 11%, 43%, 75%, and 97% RH for 28 days, followed by storage in laboratory conditions at 20 ± 2˚C. The samples used for the actual humidity measurements were cured for 28 days at 20˚C and 97 ± 5% RH. The produced humidity sensors contained 0% (Ref), 2% (S2), 4% (S4), 6% (S6), 8% (S8) and 10% (S10) of the SmartCem, calculated as the total binder weight. Before measurements were started, all sensors were kept in humidity chambers at 11%, 43%, 75% or 97% RH for 24 h. The humidity chambers consisted of sealed glass containers containing various types of saturated salt solutions. These included lithium chloride (LiCl), potassium carbonate (K_2_CO_3_), sodium chloride (NaCl) and potassium sulphate (K_2_SO_4_) which can maintain relative humidities of 11%, 43%, 75%, and 97%, respectively. A commercial humidity sensor type SHT85 produced by Sensirion was installed in each container as reference. Values measured after 24 h showed less than 5% variation within 24 h.

**Figure 3:**
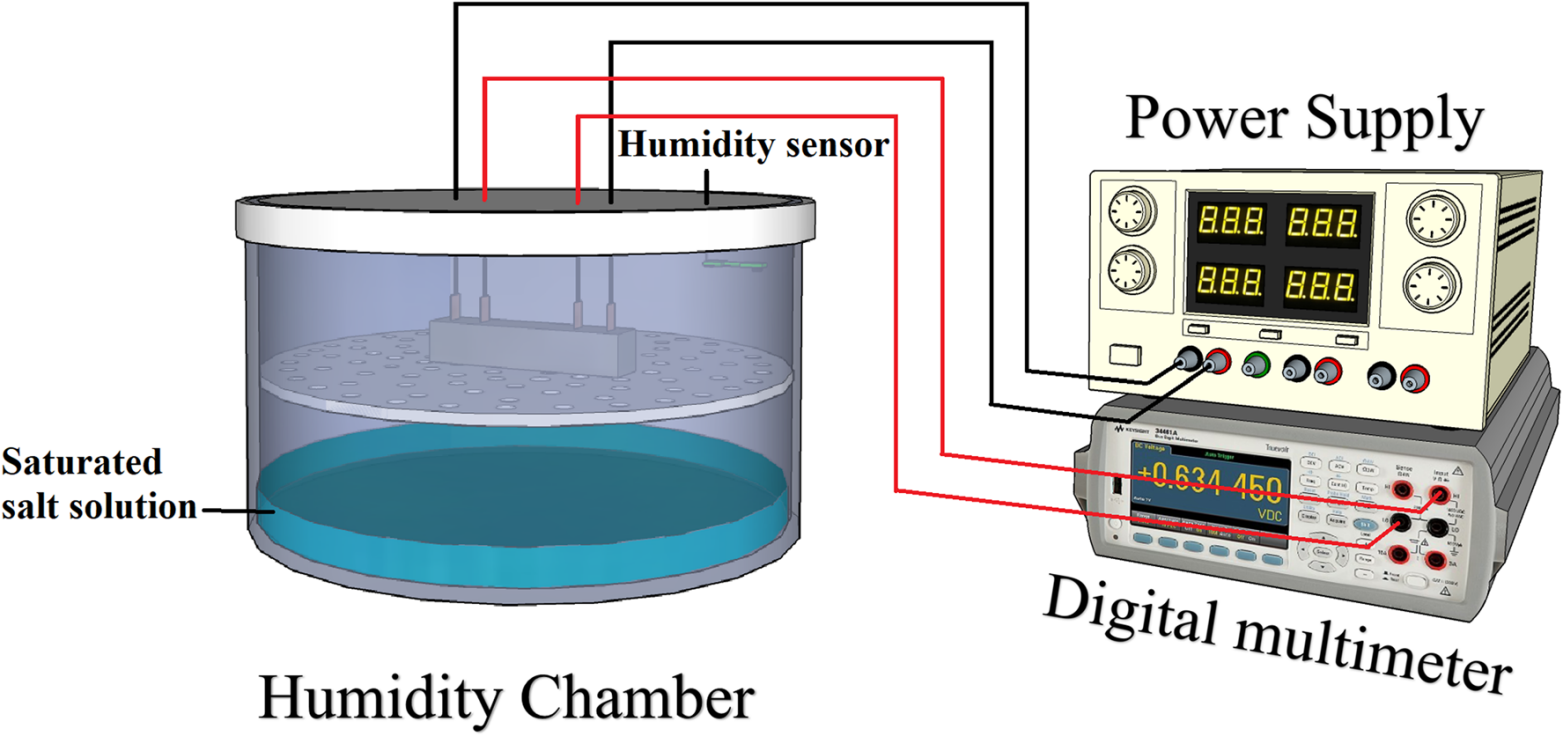
The experimental setup for electrical resistance measurements of mortar samples. This figure was created using Sketchup version 2017 (https://www.sketchup.com), Microsoft PowerPoint version Microsoft 365 (https://www.microsoft.com/en-us/microsoft-365/powerpoint).

The electrical resistance was measured using a four-probe method with a digital multimeter type Keysight 34465A. An electrical current was applied to the two outer copper electrodes while the electrical resistivity was measured on the two inner electrodes, Fig. 3. Humidity sensing was determined as a fractional change of the electrical resistivity $FCR_{Humidity}$ and calculated following the Eq. (1):

1 $$FCR_{Humidity} = {{\Delta \rho } \mathord{\left/ {\vphantom {{\Delta \rho } {\rho_{0} }}} \right. \kern-\nulldelimiterspace} {\rho_{0} }}$$

where: $\Delta \rho$ is the change of electrical resistivity, $\rho_{0}$ is the initial electrical resistivity. The electrical resistivity $\rho$ was calculated using Eq. (2):

2 $$\rho = {{R \cdot A} \mathord{\left/ {\vphantom {{R \cdot A} L}} \right. \kern-\nulldelimiterspace} L}$$

where: $R$ is the measured electrical resistance, $L$ is the internal electrode distance and $A$ is the electrode area. The sensitivity of the sensor to humidity $S_{Humidity}$ was calculated using Eq. (3)^42^**:**

3 $$S_{Humidity} = \frac{{{{\Delta R} \mathord{\left/ {\vphantom {{\Delta R} {R_{0} }}} \right. \kern-\nulldelimiterspace} {R_{0} }}}}{\Delta (\% RH)}$$

where: $\Delta R$ and $\Delta (\% RH)$ refer to the resistance change and the change of relative humidity in percentage, respectively and $R_{0}$ is the initial resistance.

## Results and discussion

There are many factors influencing electrical resistivity of cementitious matrix. These factors can be divided into two main groups. First group contains factors affecting the intrinsic electrical resistivity, especially including w/c ratio, which determines porosity, aggregate size, aggregate type, curing condition and storage condition. While the second group covers factors affecting the electrical measurement itself. For example, probe spacing, electrode contact and specimen geometry. In this research, all listed factors were kept constant, thus humidity variations can be indicated as the main affecting factor.

The effects of the humidity during curing and the sample age on the electrical resistivity of the material were determined for samples containing 100 wt.% of OPC (Ref) and a combination of 96% of OPC with 4 wt.% of the SmartCem (S4).

In general, the electrical resistivity increased with the curing age due to the consumption of water by the hydrating cement. The electrical resistivity of the S4-samples tended to be lower at all ages and exposures in comparison with the corresponding OPC samples, Table 2. The effect can be related to the creation of an additional conductive network by CNFs being present in the S4-samples. The lowest ultimate resistivity was measured for the sample mix containing 4wt.% of SmartCem (S4) and being cured at 97% RH.

**Table 2:** Effects of sample age and curing conditions on electrical resistivity.

Mix	Sample age (days)	Electrical resistivity (Ω cm)			
		Curing condition			
		11%RH	43%RH	75%RH	97%RH
Ref	1	61.24	60.57	16.61	7.06
	3	73.58	68.31	62.92	13.54
	7	254.98	245.23	188.72	22.57
	28	325.05	249.95	220.91	28.50
S4	1	49.05	46.50	13.95	7.06
	3	67.56	54.93	50.62	12.38
	7	139.21	130.41	76.38	14.16
	28	278.41	202.279	116.33	22.19

**Figure 4:**
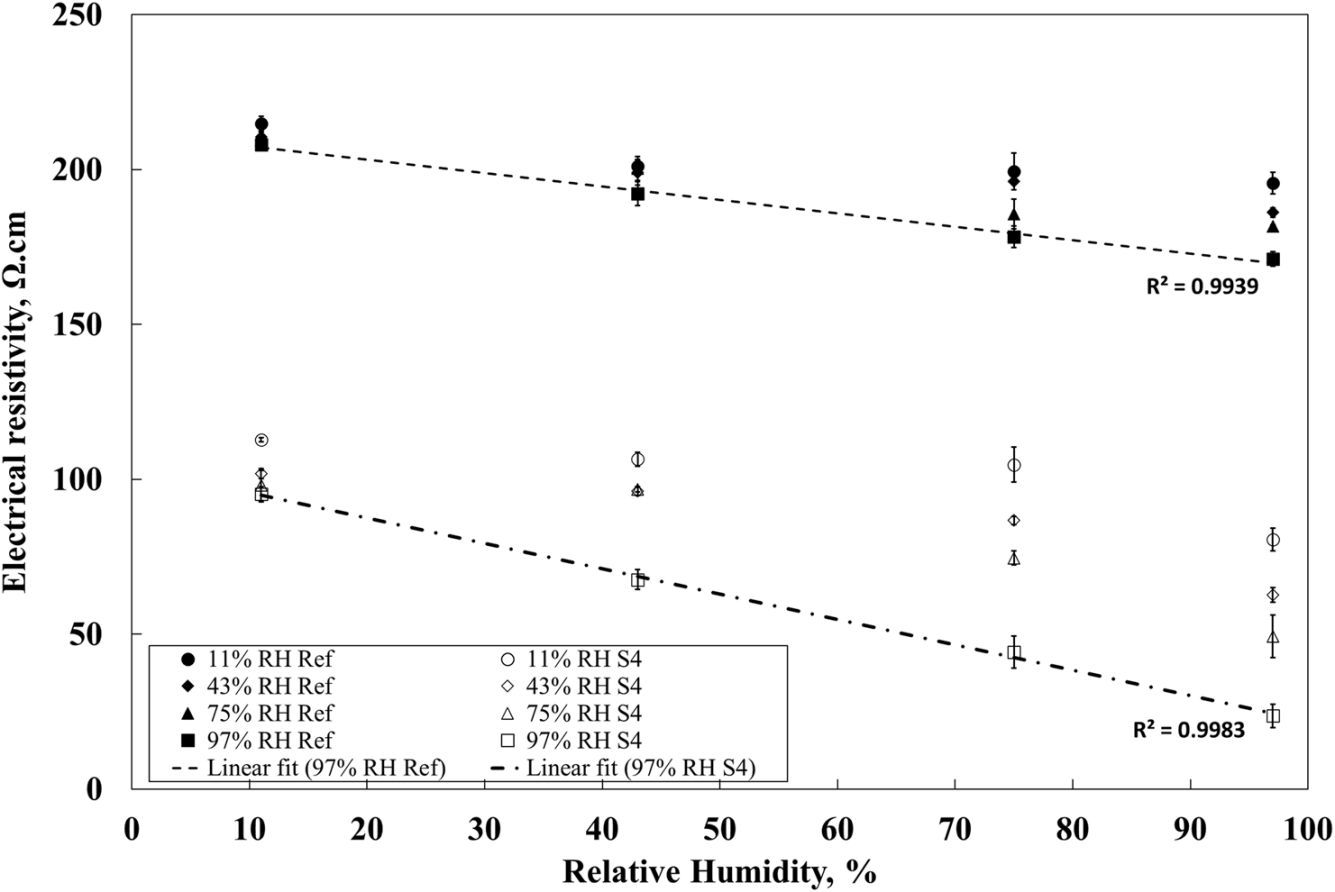
Effects of curing conditions used during production of sensors on the humidity sensing capability. Error bars represent standard errors.

In the next stage, all sensors produced from the reference mix and the S4 mix and cured at different conditions were used to determine their humidity sensing capability. Before the measurement, all produced sensors were stored for 24 h at 60% RH and 20 ± 2 ˚C. The exposure conditions included 11%, 34%, 75% and 97% RH. The resistivity measurements started 24 h later. The obtained results are shown in Fig. 4 and the calculated sensitivity of the composites are shown in Table 3.

**Table 3:** Calculated sensitivity of reference and S4 samples exposed to 11%, 43%, 75% and 97% RH.

Mix	Sensitivity, S_Humidity_ (/%RH)			
	Curing condition			
	11%RH	43%RH	75%RH	97%RH
Ref	0.00104	0.00134	0.00154	0.00206
S4	0.00126	0.00219	0.00577	0.00874

Figure 4 shows that in the case of reference samples cured at 11%RH the measured electrical resistivity was around 210 Ω cm when exposed to 11% RH and 200 Ω cm at 97% RH. A slightly higher change in resistivity was measured for samples cured at 97% RH with values of 200 and 170 Ω cm when exposed to 11% and 97% RH, respectively. Additionally, there was nearly no change of the samples containing SmartCem cured at 11% RH in the electrical resistivity until the relative humidity in the test chamber was set to 97%. In that case, the measured electrical resistivity was constant at around 110 Ω cm when exposed to 11%, 43% and 75% RH, while dropping to around 80 Ω cm at 97% RH. Samples cured at 43% and 75% RH showed a better sensitivity but only when exposed to a higher humidity, 75% and 97% RH. Only the sample cured at 97% RH could detect a humidity change in the entire measured range between 11 and 97%RH. Furthermore, for the samples cured at 97% RH the observed relation between electrical resistance and relative humidity was linear. At 11% RH the measured electrical resistivity was around 90 Ω cm while at 97% RH it decreased to 22 Ω cm.

Samples containing only the unmodified Portland cement showed a generally low sensitivity to humidity; independently of the used curing conditions. While samples containing 4 wt.% of the SmartCem showed a significantly higher sensitivity to humidity. In both cases, the lowest sensitivity was measured for samples cured at 11% RH and the highest for cured at 97% RH. The highest calculated sensitivity of 0.00874/%RH was reached for the sensor cured at 97% RH and the sensitivity of the S4 sensor significantly increased when exposing to a humidity greater than 75% RH, Table 3.

Access of moisture during curing of Portland cement affects the hydration process that controls also the developed pore structure. The pore structure and especially connectivity of capillary pores define transport of moisture within the solidified binder matrix. This in turn will ultimately affect the efficiency of a moisture sensor based on Portland cement. It has been shown that hydration of Portland cement stops when the relative humidity drops to around 80%^43^. The hydration degree of tricalcium silicate, measured after 90 days dropped from 36% to only 2% when the RH decreased from 98 to 85%^44^. Furthermore, the hydration is believed to stop when Portlandite, C-S–H and tricalcium silicate are in equilibrium which can occur at a lower RH. Limited hydration leads to a decreased amount of gel pores and coarsening of the pore structure. Tests showed a nearly three times coarser pore structure for samples cured at 80% RH in comparison with water curing^45^. These results can be directly related to the observed research trends that show that the electrical resistivity of the matrix decreased when exposed to high humidity and vice versa. Furthermore, it can be assumed that the presence of CNFs enhances the connectivity between moisture filled pore network and thus increases the electrical sensitivity of the entire system. The tunnelling effect developing between CNFs was strengthened through the presence of water molecules at the fiber–fiber and fiber-matrix interfaces^46, 47^.

The described results were used in a second set of sensor production and measurements. These sensors contained different amounts of CNFs to determine what kind of effect the CNF quantity has on the humidity-sensing capability. These sensors contained 0% (Ref), 2 wt.% (S2), 4 wt.% (S4), 6 wt.% (S6), 8 wt.% (S8) and 10 wt.% (S10) of the SmartCem. Based on the best results obtained in the first part of the study, a 97% RH was used for curing. To ensure the stability and exclude any possible effects of moisture or temperature variations on the measured electrical resistivity, the produced sensors were stored in laboratory condition for 72 h before testing.

**Figure 5:**
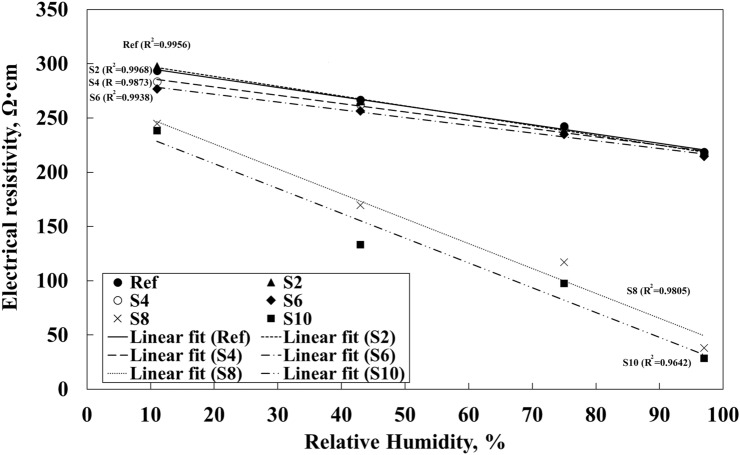
Effects of relative humidity and amount of SmartCem on electrical resistivity of produced sensors. Error bars represent standard errors.

**Figure 6:**
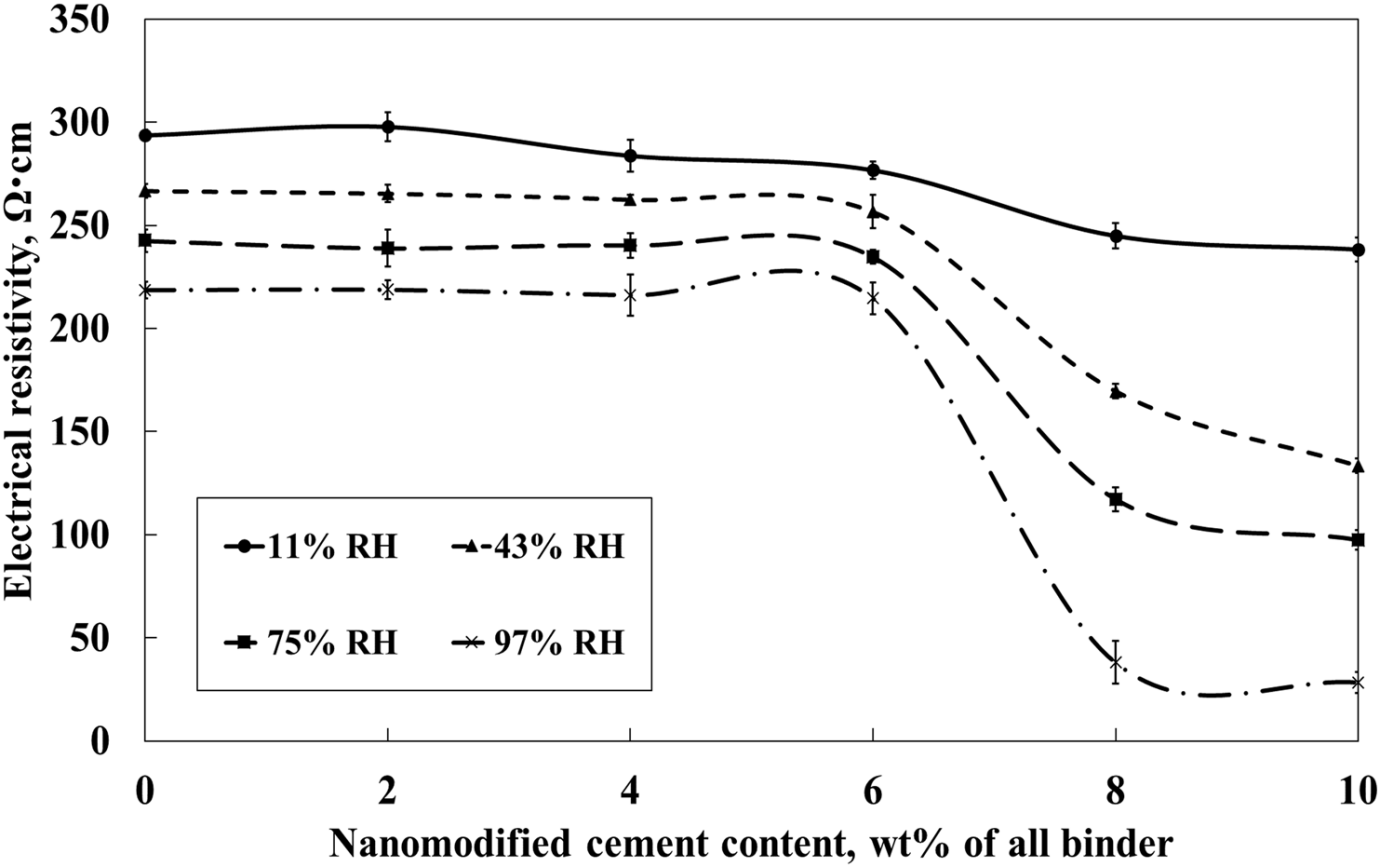
Effects of humidity and amount of nanomodified Smart cement content on the measured electrical resistivity. Error bars represent standard errors.

The test results showed a nearly linear relationship between the measured electrical resistivity and the relative humidity, Fig. 5. The R^2^ was over 0.95 in all cases, Table 4. Samples containing 0–6 wt.% of the SmartCem showed a significantly lower sensitivity of around 0.002 /%RH while samples containing 8 wt.% and 10 wt.% of the SmartCem showed a significantly higher sensitivity of 0.00982/%RH and 0.01024/%RH, respectively. The maximum humidity sensitivity of 0.01024/%RH was measured for the sensor containing 10 wt.% of the SmartCem. The measured electrical resistivity varied between 280 and 300 Ω cm at 11% RH and 230 Ω cm at 97% RH, Fig. 5. At 11% RH the measured value was around 240 Ω cm for both samples and around 300 Ω cm at 97%RH. Analysis showed that the percolation threshold related to the amount of CNFs humidity sensing was around 7 wt.% which corresponded to around 0.19 wt.% of CNFs, Fig. 6.

**Table 4:** Calculated sensitivity and R^2^ for the produced humidity sensors cured at 97% RH.

Mix	CNFs concentration (wt.% of binder)	Sensitivity of sensor, S_Humidity_ (/%RH)	R^2^
Ref	0.000	0.00277	0.9956
S2	0.054	0.00278	0.9968
S4	0.108	0.00297	0.9873
S6	0.163	0.00261	0.9938
S8	0.217	0.00982	0.9805
S10	0.271	0.01024	0.9642

Comparison between sensing capability of cementitious matrixes incorporating different types of carbon-based materials is shown in Table 5. Various types of conductive materials showed different humidity sensing capabilities. The observed differences in humidity sensitivity can be related to several factors mentioned earlier. These include the types of the used conductive fibers and their dispersion in the matrix. Matrixes studied in the present research showed significantly higher sensitivity.

**Table 5:** Sensitivity to humidity of cementitious matrixes incorporating various types of carbon-based materials.

Reference	Amount of carbon-based materials (wt.% of cement)	Humidity range	Measurement method	Calculated change of the electrical resistivity (%)	Calculated humidity sensitivity (/%RH)
Xiaoming^48^	25% graphite	20–60%	Four-probe	~ 9.09%	~ 0.00227
Carisio et al.^49^	0.20% MWCNT	0–90%	Four-probe	~ 49.33%	~ 0.00548
Carisio et al.^49^	0.35% MWCNT	0–90%	Four-probe	~ 49.64%	~ 0.00551
Present result S10	0.271% CNF 10% SmartCem	11–97%	Four-probe	88.06%	0.01024

**Figure 7:**
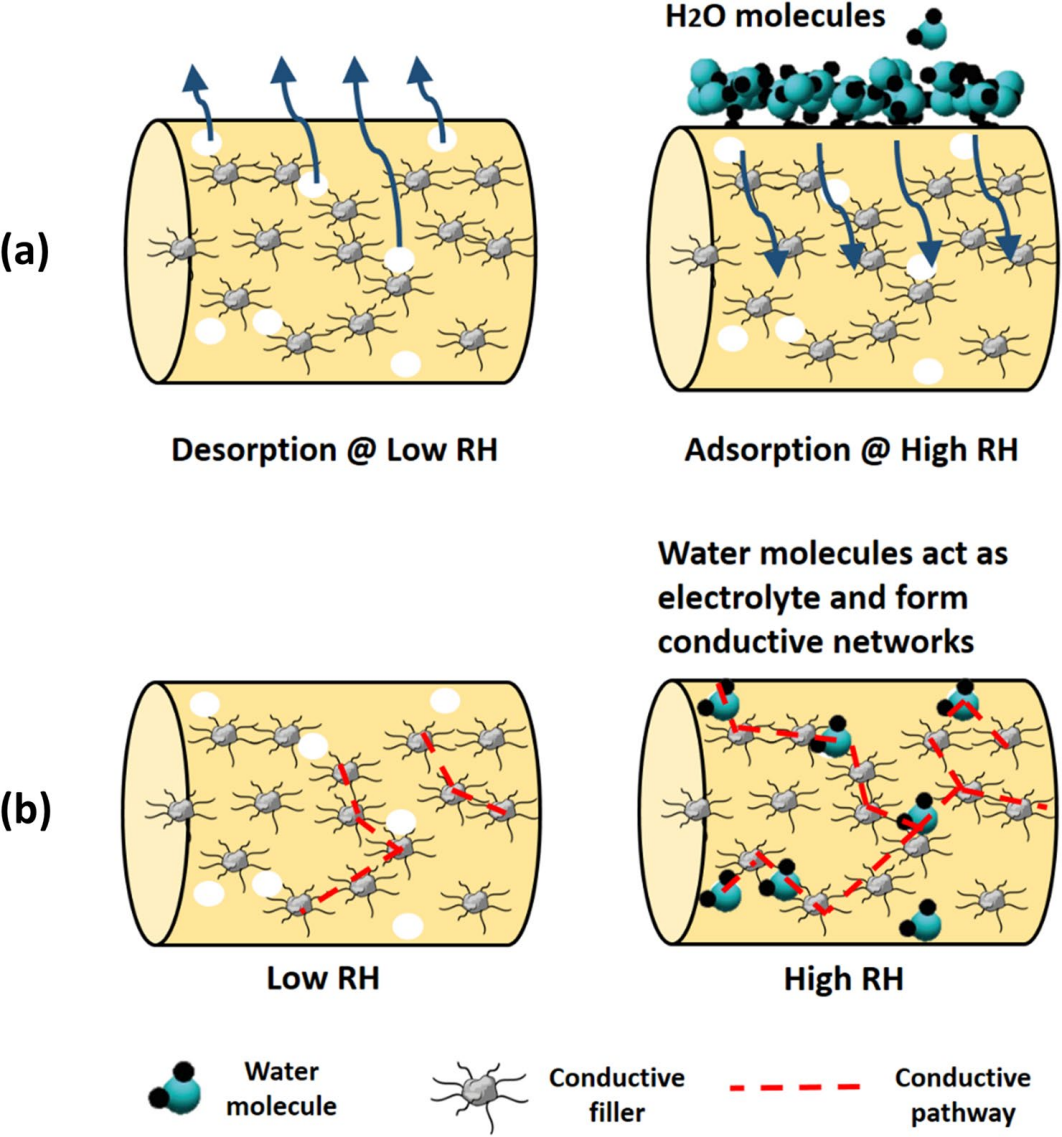
Schematic description of the effect of humidity on **(a)** the microstructure of the matrix **(b)** conductive paths in the matrix. This figure was created using Sketchup version 2017 (https://www.sketchup.com), Microsoft PowerPoint version Microsoft 365 (https://www.microsoft.com/en-us/microsoft-365/powerpoint).

Summarising, the humidity sensing mechanism of the developed SmartCem sensors is related to an alteration of the electrical resistivity. Absorption or desorption of water molecules due to variation of RH in this study change the interconnection between matrix–matrix, fiber–fiber and fiber-matrix resulting in a change in the ultimate electrical resistivity of sensors. For example, water molecules in gaseous state adsorb on the external surface of the sensor and later diffuse into the matrix due to capillary condensation, Fig. 7a. The water vapour condenses into water and gradually fills up the abundant pores. This generates additional “bridges” between existing electrically conductive paths. Consequently, different amounts of water vapour will result in different ultimate electrical resistivity of the sensor, which can be measured, Fig. 7b ^50^. A comprehensive micromechanical model was proposed by Jang et al.^51^ to predict the effective electrical conductivity of cementitious matrix containing carbon-based filler. This model confirmed that moisture affects the sensing of smart cementitious-based composite.

## Conclusion

The study aimed to determine the sensitivity of novel sensors to variations in the humidity. These humidity sensors are based on mortars containing various amounts of the nanomodified Portland cement (SmartCem). It was found that the electrical resistivity of the sensors tended to increase with longer wet curing time due to the alternation of the microstructure and hydration processes. Samples cured at 97% RH showed the highest sensitivity with the sensitivity value reaching 0.01024/%RH. According to the percolation theory, the percolation threshold amount of the nanomodified cement using for humidity monitoring is estimated at 7 wt.% of the SmartCem (~ 0.19 wt.% of CNFs). The humidity sensitivity of the nanomodified cement was related to the intrinsic electrical property, water absorption property, the connectivity as well as the amount of the nanomodified cement to change the contact point between fiber–fiber and fiber-matrix due to the presence of water vapour in the air.
